# COVID-19: A Multidisciplinary Review

**DOI:** 10.3389/fpubh.2020.00383

**Published:** 2020-07-29

**Authors:** Nour Chams, Sana Chams, Reina Badran, Ali Shams, Abdallah Araji, Mohamad Raad, Sanjay Mukhopadhyay, Edana Stroberg, Eric J. Duval, Lisa M. Barton, Inaya Hajj Hussein

**Affiliations:** ^1^Geriatric Division, Department of Internal Medicine, Beaumont Health System, Royal Oak, MI, United States; ^2^Department of Internal Medicine, Wayne State University School of Medicine, Detroit, MI, United States; ^3^Department of Emergency Medicine, Beaumont Health System, Royal Oak, MI, United States; ^4^Department of Diagnostic Radiology, Memorial Sloan Kettering Cancer Center, New York, NY, United States; ^5^Department of Cardiology, Henry Ford Health System, Detroit, MI, United States; ^6^Department of Pathology, Cleveland Clinic, Cleveland, OH, United States; ^7^Office of the Chief Medical Examiner, Oklahoma City, OK, United States; ^8^Department of Foundational Medical Studies, Oakland University William Beaumont School of Medicine, Rochester, MI, United States

**Keywords:** SARS-CoV-2, COVID-19, coronavirus, respiratory infection, pandemic, global health

## Abstract

Severe acute respiratory syndrome coronavirus-2 (SARS-CoV-2) is a novel coronavirus that is responsible for the 2019–2020 pandemic. In this comprehensive review, we discuss the current published literature surrounding the SARS-CoV-2 virus. We examine the fundamental concepts including the origin, virology, pathogenesis, clinical manifestations, diagnosis, laboratory, radiology, and histopathologic findings, complications, and treatment. Given that much of the information has been extrapolated from what we know about other coronaviruses including severe acute respiratory syndrome coronavirus (SARS-CoV) and Middle East respiratory syndrome coronavirus (MERS-CoV), we identify and provide insight into controversies and research gaps for the current pandemic to assist with future research ideas. Finally, we discuss the global response to the coronavirus disease-2019 (COVID-19) pandemic and provide thoughts regarding lessons for future pandemics.

## Introduction

The world has witnessed numerous epidemics and pandemics that have affected thousands to millions of lives. Despite our advances in medicine and research, we continue to be challenged with new pathogens that pose a threat to human lives, global economic security, and the healthcare system. Severe acute respiratory syndrome coronavirus-2 (SARS-CoV-2) is a novel coronavirus that was first identified in Wuhan, Hubei province, central China, and is responsible for the 2019-20 pandemic.

SARS-CoV-2 is the seventh coronavirus to date that is known to infect humans. This has been possible by frequent cross-species infections and occasional spillover events (1). Two of these previously identified coronaviruses were responsible for major epidemics in the past two decades; Severe Acute Respiratory Syndrome Coronavirus (SARS-CoV) also originating from China in 2002–2003 and the Middle East Respiratory Syndrome Coronavirus (MERS-CoV) originating from the Middle East in 2012 (2, 3). All three of these coronaviruses are considered zoonotic in origin and have the ability to cause severe and fatal illness in humans (3, 4). Unfortunately, given their large genetic diversity and the frequent recombination of their genomes coupled with the increase in human-animal interface activities due to modern agricultural practices, novel coronaviruses are likely to continue to develop and cause periodic seasonal spreads (3).

Here, we provide a multidisciplinary review of the current literature involving the SARS-CoV-2 virus. We review the origin of the virus, the course of disease, the therapeutic investigations, and the global response. Specifically, we discuss the pathogenesis, histopathology, virology, and immune response. We also examine the clinical manifestations, diagnosis, laboratory and radiology findings, in addition to common complications. This is followed by a briefing on the existing literature regarding adjunctive therapies and ongoing trials. Finally, we discuss the global response to the coronavirus disease-2019 (COVID-19) pandemic and the lessons learned for future pandemics.

## Timeline to Pandemic

Though the specific date varies according to different reports, it is postulated that the outbreak started in Wuhan around December 12, 2019, when multiple patients presented with similar clinical symptoms including fever, cough, dyspnea, and atypical pneumonia (3). On December 29, four cases of “pneumonia of unknown etiology” were officially reported by local hospitals using a surveillance mechanism that was established following the 2002–2003 SARS epidemic with the aim of allowing timely identification of novel pathogens. All four of these cases were thought to have a connection to a local seafood market, Huanan Seafood Market, which sold live non-aquatic wild animals (5, 6).

In an attempt to identify the causative pathogen, three bronchoalveolar lavage fluid samples from one patient with “pneumonia of unknown etiology” were collected and sent for identification on December 30. Whole genome sequencing and bioinformatic analyses revealed that the virus features were typical of the beta-coronavirus 2B lineage of the coronavirus (7). Additionally, the genome of the novel virus was found to be 96% identical to the bat SARS-like coronavirus strain BatCov RaTG13, a bat coronavirus detected in Rhinolophus affinis from Yunnan province (2).

On December 31, the Chinese authorities alerted the World Health Organization (WHO) of these cases. Due to the continued connection of emerging cases to the Huanan Seafood Market, the market was eventually closed on January 1, 2020 for sanitization. On January 6, the Chinese Center for Disease Control and Prevention (China CDC) activated a Level 2 emergency response. On January 8, a novel coronavirus was officially announced to be the cause of the outbreak and on January 10, the first genome sequence for the virus was released by China CDC. The novel virus was initially called the 2019 novel coronavirus (2019-nCoV). The WHO subsequently changed the name to SARS-CoV-2 on February 11 due to its vast resemblance to SARS-CoV (8).

**Figure 1:**
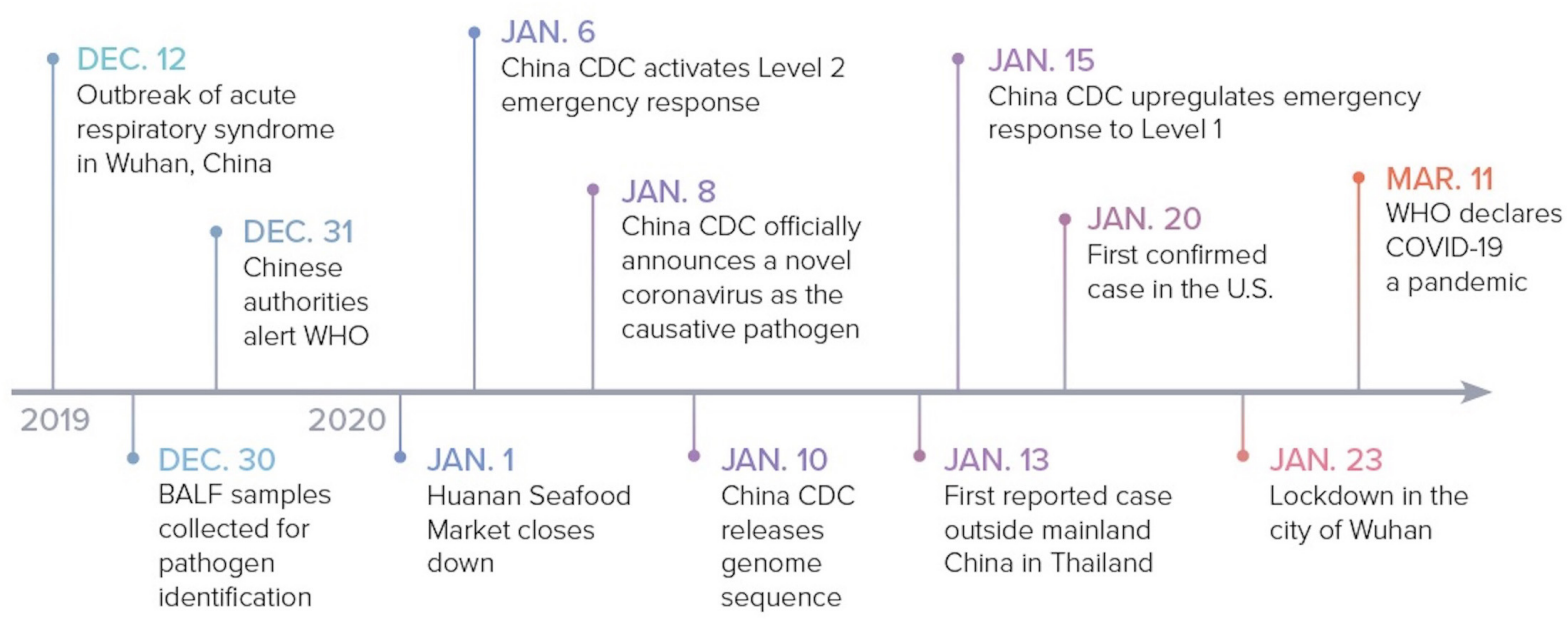
COVID-19: timeline to pandemic. The sequence of events from the outbreak in Wuhan, China to the declaration of the COVID-19 pandemic. BALF, bronchoalveolar lavage fluid.

The first case reported outside of China was on January 13 in Thailand. China CDC upgraded the emergency response to Level 1 on January 15 (9). On January 20, the CDC confirmed the first case in the United States (U.S.) in Washington state, which was linked to recent travel from Wuhan (10). Due to the continued surge of new cases, the Chinese government ordered a complete lock down of Wuhan on January 23. By January 30, the WHO declared a global health emergency and COVID-19 was declared a pandemic on March 11, 2020 (9) (Figure 1).

As of the beginning of June, there were more than 7 million confirmed cases of COVID-19 with more than 400 thousand deaths globally. This pandemic has spread to more than 200 countries, areas, or territories across the world (11). In comparison, SARS-CoV spread to 12 countries including the U.S. with a total of 8,096 confirmed cases and 774 deaths until it was contained in 2003 (12). MERS-CoV spread to 27 countries, including the U.S., with a total of 2,494 confirmed cases and 858 deaths (13) (Table 1).

**Table 1:** Comparison between SARS-CoV-2, MERS-CoV, and SARS-CoV.

	**SARS-CoV-2**	**MERS-CoV**	**SARS-CoV**
Pandemic/ epidemic year	2019-Present	2012	2002–2003
Coronavirus subfamily	Beta–Coronavirus	Beta–Coronavirus	Beta–Coronavirus
Natural reservoir	Bat	Bat	Bat
Intermediate host	Pangolin	Dromedary camel	Palm civets
Origin	Wuhan, China	Arabian Peninsula	Guangdong, China
Country spread	>180	27	26
Total cases to date	>7,000,000	2,494	8,096
Total deaths to date	>400,000	858	774
Total cases in the U.S. to date	>1,900,000	2	27
Case fatality rate^*^	1–7.2%	34.4%	9.6%

* *Case fatality rate varies in different countries depending on different testing strategies, definition of COVID-19 related deaths, and population age. Numbers are subject to change with the ongoing COVID-19 pandemic (1, 11–14)*.

## The Origin of SARS-CoV-2

It is crucial to identify the origin, hosts, and evolutionary pathway of the causative pathogen of a pandemic to be able to implement proper control measures and help prevent future pandemics. Unfortunately, the exact origin of SARS-CoV-2 remains unclear so many theories have been proposed based on information stemming from SARS-CoV.

After the SARS epidemic in 2002, bats were first recognized to be hosts for coronaviruses and interest grew in identifying other potential mammal hosts (15). The majority of early cases of SARS occurred in patients with close contact to animals including market palm civets. Soon afterwards, SARS-CoV was cultivated from caged Himalayan palm civets from live wild markets in Guangdong, China. Upon further investigation, with the discovery of many coronaviruses phylogenetically related to SARS-CoV in bats from different provinces in China and other countries, bats were believed to be the natural reservoir for SARS-CoV, and the palm civet was a possible intermediate host. It was likely that the virus acquired multiple mutations in the market palm civets before spillover to humans (1, 6). Bats were also believed to be the natural reservoir for MERS-CoV and dromedary camels were thought to be the intermediate hosts. Bats have since been discovered to be the hosts of a minimum of 30 coronaviruses with available complete genome sequences (15). This may be an underestimation since many more coronaviruses may exist that have yet to be identified or sequenced.

As previously stated, SARS-CoV-2 has been found to be 96% identical at the whole genome level to the bat SARS-like coronavirus strain BatCov RaTG13, making it likely that bats served as reservoir hosts. With many theories not supportive of direct spillover from bats to humans, further investigation was conducted. Pangolins were then reported as potential intermediate hosts after samples were analyzed from Malytan pangolins, an endangered species illegally trafficked into southern China for use in old-fashioned Chinese medicine and as a food source. These were obtained from Guangdong and Guangxi, China during an anti-smuggling operation. Samples from the pangolins showed new coronavirus genomes with 85.5–92.4% resemblance to SARS-CoV-2. More remarkable was the 97.4% amino acid similarity in the receptor binding domain (RBD) of coronavirus genomes from pangolins compared to SARS-CoV-2. In comparison, the Bat CoV RaTG only had 89.2% amino acid similarity in the RBD with SARS-CoV-2. Up until now, bats and pangolins are the only two mammals known to be infected by SARS-CoV-2-related coronaviruses (6, 16) (Table 1).

## Viral Morphology

Coronaviruses are enveloped, positive single-stranded RNAs with the largest known RNA genome ranging from 26 to 32 kilobases in length (8, 17). They are spherical virions with a core shell and a surface that resembles a solar corona based on its surface protein projections, hence their name (Latin: corona = crown) (8). There are four main subfamilies; alpha-, beta-, gamma- and delta- coronaviruses.

Alpha- and beta-coronaviruses originate from mammals, mainly bats, and are thought to cause more severe and fatal diseases in humans, while gamma- and delta-viruses mainly originate from birds and pigs and are thought to cause asymptomatic or mild disease in humans (8).

**Figure 2:**
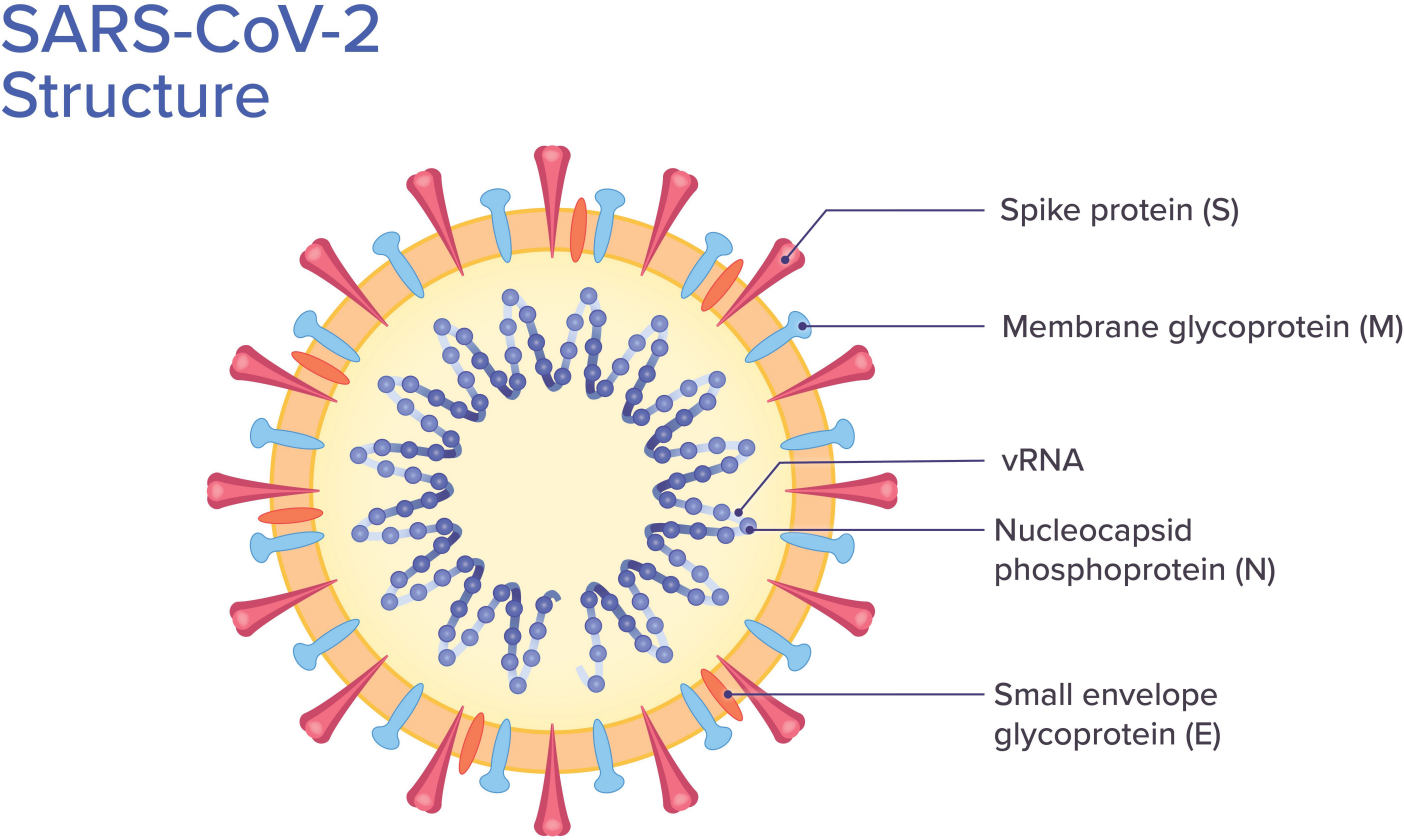
SARS-CoV-2 structure. Viral structure with its protein components and viral RNA (vRNA).

SARS-CoV-2 belongs to the beta-coronavirus group, which also includes MERS-CoV and SARS-CoV. The latter shares ~75–80% of its viral genome with SARS-CoV-2 (8, 18). Beta-coronaviruses have three important envelope proteins: Spike (S) protein, Membrane (M) protein, and Envelope (E) protein. S protein mediates viral attachment to the cell membrane receptor, membrane fusion, and ultimately viral entry into the host cell. M protein, the most abundant membrane protein, together with E protein are responsible for the coronavirus membrane structure. Another component of the beta-coronavirus is the N protein, which is the protein component of the helical nucleocapsid that includes the genome RNA (19) (Figure 2).

## Mode of Transmission

According to current evidence, the WHO reports that SARS-CoV-2 transmission occurs via respiratory droplets and contact routes. Droplet transmission occurs through direct contact when a person is exposed to infective respiratory droplets when they are within 1 m of someone with respiratory symptoms including coughing and sneezing. Being within this distance puts the individual at risk of having their mucous membranes, including their mouth, nose and eyes, exposed to the droplets. Transmission can also occur through indirect contact by way of fomites on surfaces in the immediate environment around the infected person. Airborne transmission may be possible when aerosol-generating procedures are performed including endotracheal intubation, cardiopulmonary resuscitation, administration of nebulized treatments, and others (20).

Transmission of the virus can occur in the pre-symptomatic incubation period. A study in a nursing home showed that more than half of the residents with positive test results for SARS-CoV-2 infection were pre-symptomatic and most likely contributed to transmission (21). Asymptomatic transmission (i.e., in patients who never develop symptoms) can also occur as suggested in some studies (22, 23).

In terms of infectivity, the basic reproductive number (R0), which is defined as the expected average number of additional infectious cases that one infectious case can generate, was thought to range from 2.2 to 2.7 for SARS-CoV-2 infection in the early stages of the epidemic in China. This means that one person infected with SARS-CoV-2 can spread the infection to ~2.2–2.7 people (5, 24). This number is subject to change with the progression of this pandemic, especially following the introduction of better control measures (5). The R0 for SARS-CoV was estimated to be around 3 after critically comparing various independent studies (25). However, the SARS-CoV outbreak was better controlled compared to the current pandemic due to successful isolation of infected patients (5). The R0 for MERS-CoV was estimated to range from 2 to 5 in Saudi Arabia and South Korea (26).

## Pathogenesis

Although the pathogenesis of SARS-CoV-2 is not clearly understood, information regarding viral replication and pathogenesis can be extracted from what we know about other beta-coronaviruses (SARS-CoV and MERS-CoV) due to their similarities to SARS-CoV-2.

### Direct Viral Injury

SARS-CoV-2 binds to epithelial cells in the oral and nasal cavities and can also migrate further down the respiratory tract into the conducting airways. SARS-CoV has been shown to infect primary ciliated cells in the conducting airway and therefore, it has been hypothesized that the same occurs with SARS-CoV-2. About 80% of the infected patients will have a mild course limited to the upper and conducting airways (27).

**Figure 3:**
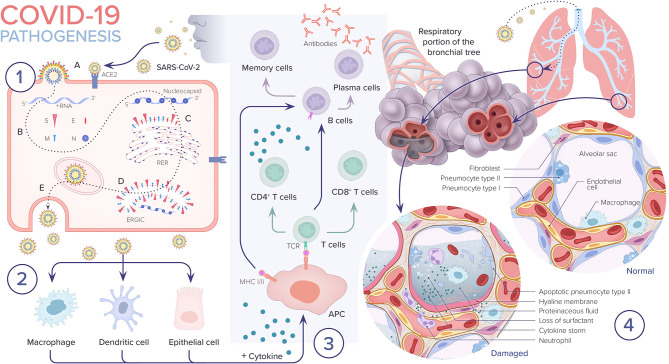
COVID-19 pathogenesis. 1. A. SARS-CoV-2 enters the epithelial cell either via endocytosis or by membrane fusion through binding to ACE2 receptor and releasing its RNA into the cytoplasm. B. Viral RNA uses the cell's machinery to translate its viral non-structural and structural proteins and replicate its RNA. C. Viral structural proteins S, E, and M assemble in the rough endoplasmic reticulum (RER). D. Viral structures and nucleocapsid subsequently assemble in the endoplasmic reticulum golgi intermediate (ERGIC). E. New virion packed in golgi vesicles fuse with the plasma membrane and get released via exocytosis. 2. SARS-CoV-2 infection induces inflammatory factors that lead to activation of macrophages and dendritic cells. 3. Antigen presentation of SARS-CoV-2 via major histocompatibility complexes I and II (MHC I and II) stimulates humoral and cellular immunity resulting in cytokine and antibody production. 4. In severe COVID-19 cases, the virus reaches the lower respiratory tract and infects type II pneumocytes leading to apoptosis and loss of surfactant. The influx of macrophages and neutrophils induces a cytokine storm. Leaky capillaries lead to alveolar edema. Hyaline membrane is formed. All of these pathological changes result in alveolar damage and collapse, impairing gas exchange.

The virus can progress even further and can infect the alveolar type II pneumocyte cells, similar to SARS-CoV. It has been shown that SARS-CoV are released in large numbers from infected type II pneumocytes and cause cell apoptosis. Type II pneumocyte cells normally comprise 10–15% of total lung cells. They produce surfactant, which is responsible for the maintenance of surface tension in alveolar walls. These cells are also responsible for maintaining the lung epithelium after injury through epithelial regeneration (28). Therefore, as replicated viral particles are released from the cell and move on to infect more type II pneumocytes, the resulting apoptosis eventually causes diffuse alveolar damage and impaired gas exchange, which is hypothesized to lead to acute respiratory distress syndrome (ARDS). A similar mechanism is postulated for SARS-CoV-2 (27) (Figure 3).

### Viral Replication Cycle

SARS-CoV-2 has been shown to use the angiotensin-converting enzyme 2 (ACE2) receptor for cell entry, similar to SARS-CoV (2). Through the examination of human tissue specimens, ACE2 receptors have been found in various organs and cells including the nasopharynx, nasal and oral mucosa, small intestine, colon, kidney, liver, vascular endothelium, and epithelial cells of lung alveoli (mainly type II pneumocytes) (29).

The RBD in the S protein of SARS-CoV-2 specifically recognizes its host ACE2 receptor. The viral RBD region is made of 394 glutamine residues and is recognized by 31 lysine residues of the human ACE2 receptor. Previous studies revealed that host susceptibility to SARS-CoV infection is mainly determined by the affinity between the host ACE2 receptor and the viral RBD in the early viral attachment phase. It is thought that this mechanism is likely similar in SARS-CoV-2 and that a genetic recombination event in the RBD region of SARS-CoV-2 may be the cause of its higher transmission rate as compared to SARS-CoV (30).

After cell entry, the viral RNA positive sense genome is released into the cell cytoplasm and undergoes translation and replication forming progeny genomes and sub-genomic mRNAs. The latter translates into membrane proteins, N protein, and a variety of accessory proteins (19). SARS-CoV has its own central enzyme called the RNA-dependent RNA polymerase, which, along with other viral and cellular proteins, composes the main replication complex responsible for replicating the viral genome (31).

The formed membrane proteins (S, M, and E) are then inserted into the rough endoplasmic reticulum (RER) and are transported to the endoplasmic reticulum-golgi intermediate compartment (ERGIC). N proteins along with genomic RNA then form nucleocapsids, which fuse into the ERGIC. Finally, the pathogen gets transported to the plasma membrane and is exported out of the cell via exocytosis (19, 32) (Figure 3).

### Immune System Activation and Cytokine Storm Syndrome

When the virus enters the cell, its antigen is presented by the antigen-presenting cells (APCs) such as dendritic cells and macrophages. This leads to the activation of the body's humoral and cellular immunities, which are mediated by virus-specific B and T cells (32, 33). Antigen presentation occurs via major histocompatibility complexes (MHC; or human leukocyte antigen (HLA) in humans) present on the surface of APCs and recognized by virus-specific cytotoxic T lymphocytes (CTLs). There are two major classes of MHCs involved in antigen presentation: MHC 1 and MHC II. SARS-CoV mainly depends on MHC I molecules. Unfortunately, the evidence regarding antigen presentation in SARS-CoV-2 is lacking and most of the information is extrapolated from prior studies done on SARS-CoV and MERS-CoV. Studies have shown that different HLA genotypes may be responsible for differences in host susceptibility to the virus and therefore, severity of disease. Patients infected with SARS-CoV with HLA-B^*^46:01 genotypes were shown to have more severe disease compared to those with different genotypes. This has not been clinically validated in studies on SARS-CoV-2 as of yet (32).

Once CD4+ T cells, also known as helper T cells, are activated, they cause the release of cytokines and chemokines (Figure 3). If exaggerated, this leads to the development of cytokine storm syndrome. The exact mechanism by which the immune system response to a viral infection can lead to cytokine storm syndrome is not completely understood. It has been shown that certain viruses are capable of altering the immune response to infection predisposing the host to develop a cytokine storm. Cytokine storm syndrome has been described in prior viruses including SARS-CoV, dengue and influenza virus. It remains a challenge to understand why some patients develop a cytokine storm while others do not. Research has shown that genetic polymorphisms, for example changes in the toll-like receptors (TLR), may play an important role in affecting host responses to certain infections, ultimately leading some to develop a cytokine storm (34).

Acute lung injury, including its severe form ARDS, is a common consequence of cytokine storm syndrome. This has been shown to occur in patients with SARS-CoV-2 infection with the development of diffuse lung injury, inflammation, and fluid buildup, which can ultimately lead to death. ARDS is also a common immunopathological event in both SARS-CoV and MERS-CoV (32). A study done in Wuhan, China noted that patients infected with SARS-CoV-2 had high amounts of pro-inflammatory cytokines and chemokines in their plasma. Critically ill patients who required intensive care unit (ICU) admission were found to have higher concentrations of cytokines in their plasma as compared to those with milder illness, suggesting that cytokine storm was connected to disease severity (35). Similarly, patients with severe MERS-CoV and SARS-CoV infections showed higher levels of interleukin-6 (IL-6), a pro-inflammatory cytokine, and chemokines in their serum compared to those with mild disease (32).

IL-6 has received special attention. IL-6 plays a key role in cytokine storm syndrome. It has both anti-inflammatory and pro-inflammatory effects. IL-6 binds to its transmembrane and soluble receptors, which result in the activation of the inflammatory response potentially leading to cytokine storm (36). IL-6 levels have been shown to be ~2.9 folds higher in patients with complicated disease, mainly those requiring ICU admission, compared to those with mild disease, with higher levels associated with a higher incidence of death (37).

### Immunity

Individuals who become infected with SARS-CoV-2 produce antibodies against the virus. Most studies show that patients infected with SARS-CoV-2 develop antibody titers at days 10 to 15 after symptom onset. Based on preliminary evidence, these antibodies may have a protective role, however this is yet to be established (38, 39) An observational cohort study in Hong Kong showed a correlation between antibody titers detected by ELISA and virus neutralization titers (38). However, another study involving 175 patients who recovered from SARS-CoV-2 showed that a proportion of them developed very low antibody titers (below the detectable level) despite recovering from the disease. Therefore, further studies are needed to establish if antibody titers determine the likelihood to recover from disease (39). Further studies are needed to understand whether antibody titers reflect immunity, and if so, at what level and for how long.

### RAAS Inhibitors and COVID-19

**Figure 4:**
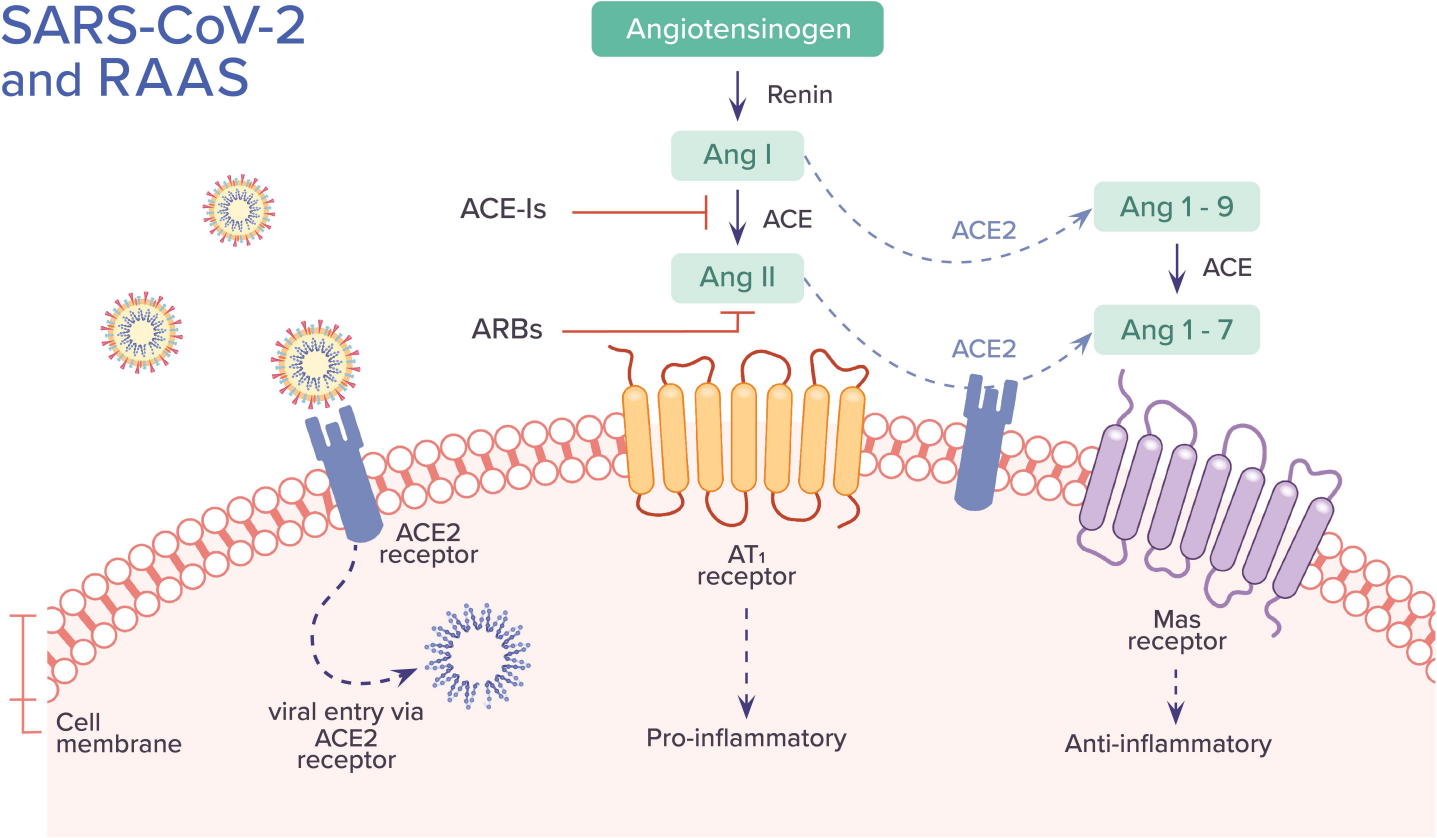
The association between SARS-CoV-2 and the Renin-Angiotensin-Aldosterone System (RAAS). SARS-CoV-2 binds to ACE2 receptor and enters into the cell. It has been hypothesized that this process leads to down-regulation of surface ACE2, resulting in unopposed angiotensin II buildup and activity, leading to a pro-inflammatory cascade. Alternative hypotheses are further described in the text. The uncertainties regarding the role of ACE-Is and ARBs in COVID-19 are also discussed. Ang, angiotensin; ACE, angiotensin-converting enzyme; AT1, angiotensin II type 1 receptor.

SARS-CoV and SARS-CoV-2 are involved with the renin-angiotensin-aldosterone system (RAAS) through ACE2, the enzyme that functions as a receptor for both viruses and also physiologically counters RAAS activation (40). Within RAAS, angiotensin I is converted to angiotensin II by ACE. Angiotensin II mediates vasoconstrictive and pro-inflammatory effects through angiotensin II type 1 receptor (AT_1_R). ACE2, on the other hand, converts angiotensin II to angiotensin I-7, which binds to Mas receptor and facilitates numerous functions including vasodilation and anti-inflammatory effects. ACE2 also converts angiotensin I to angiotensin I-9, which can be further converted by ACE to angiotensin I-7. ACE2 limits the adverse vasoconstrictor and pro-inflammatory properties of angiotensin II by degrading it and by the formation of angiotensin I-7, counteracting its action. ACE inhibitors (ACE-Is) block the conversion of angiotensin I to angiotensin II. Angiotensin receptor blockers (ARBs) inhibit the binding of angiotensin II to AT_1_R and angiotensin II type 2 receptor (AT_2_R); its affinity for AT_1_R, the main pathway by which angiotensin II exerts its pro-inflammatory effects, is 1,000 times greater than AT_2_R (41) (Figure 4).

A large proportion of COVID-19 patients have preexisting hypertension. Also, patients with more severe illness are more likely to have hypertension than those with mild illness. This has sparked concerns that the RAAS inhibitors used for medical management of hypertension may somehow be contributing to poor outcomes through their effect on ACE2 (40). Conflicting data exists regarding the effect of RAAS inhibitors on levels and expression of ACE2 in various human tissues (41). In studies involving patients with a variety of cardiac conditions, plasma ACE2 activity was not higher among patients taking ACE-Is or ARBs when compared to patients not treated with these medications. On the other hand, in a longitudinal cohort study in Japan involving patients with hypertension, those who received long-term treatment with the ARB olmesartan had higher urinary ACE2 levels than control patients. However, the same findings were not present among patients using ARBs other than olmesartan or the ACE-I enalapril (40). Data showing the effects of RAAS inhibitors on lung-specific expression of ACE2 specifically are lacking (42).

It is unclear whether RAAS inhibitors increase, decrease, or have no effect on levels and expression of ACE2. It is also uncertain whether increased ACE2 would have protective or detrimental effects. It is thought that increased ACE2 would be detrimental as it would facilitate greater entry of SARS-CoV-2 into the cell causing higher disease virulence. Instead, some postulate that increased ACE2 may have a beneficial role in SARS-CoV-2 infection by attenuating virus-induced lung injury due to the vasodilator and anti-inflammatory role of the ACE2 pathway (42). Finally, others have proposed that SARS-CoV-2 entry into the cell downregulates ACE2 expression based on studies done *in vitro* in cultured cells, which showed that viral infection and replication contributed to reduced membrane ACE2. Down-regulation of ACE2 activity may be detrimental as it would cause unopposed accumulation of angiotensin II leading to the organ injury seen in COVID-19 (40).

The uncertainties outlined above make it difficult to offer guidance regarding the use of these medications in patients with COVID-19. The results of a retrospective Chinese study in Wuhan involving 1,178 patients hospitalized with COVID-19 showed that the frequency of severe disease, ARDS, and mortality did not differ in those using ACE-Is or ARBs compared to those not using these medications (43). Also, there is clear potential for harm associated with the withdrawal of RAAS inhibitors in patients in otherwise stable condition. RAAS inhibitors have well-established benefits in protecting the myocardium, and their withdrawal causes clinical decompensation in high-risk patients as has been shown in multiple studies. For example, in the Quinapril Heart Failure Trial, withdrawal of quinapril in patients with chronic symptomatic heart failure resulted in a progressive decline in clinical status. Among patients dealing with an unstable clinical status and ongoing myocardial injury due to COVID-19, withdrawal of RAAS inhibitors may pose an even higher risk (40).

Therefore, societies including the American College of Cardiology (ACC) have supported the continuation of RAAS inhibitors in patients in otherwise stable condition who are at risk for, are being evaluated for, or have been diagnosed with COVID-19 (40). The ACC advises that patients should continue taking RAAS inhibitors for conditions such as heart failure, hypertension, or ischemic heart disease, and that if COVID-19 occurs, “individualized treatment decisions should be made according to each patient's hemodynamic status and clinical presentation” (44).

## Histopathology

Compared to the robust clinical literature, there are relatively few published reports on the histopathology of COVID-19, none of which are large series (45–52). Published reports as of the time of this writing (April 25, 2020) are summarized in Table 2. The first description of COVID-19 histopathology came from China and consisted of a single case report based on post-mortem core biopsies of the lung, liver and heart (52). This was followed by another publication from China on the pathologic findings in two lobectomies for lung cancer in which the patients developed symptoms of COVID-19 after surgery (49). The authors postulated that the (rather non-specific) findings observed in the lungs possibly represented early COVID-19 pathology. On April 10, 2020, the first findings of complete autopsies in the English literature were described by Barton et al. from the United States (46).

**Table 2:** Histopathology of COVID-19 in peer-reviewed English language journals.

**Date**	**First author (country)**	**Specimen type**	**No. of cases**	**Main findings**	**DAD**	**Thrombi**
Feb 18, 2020	Xu Z (China) (52)	Post-mortem biopsies of lung, liver, heart	1	DAD	Yes	None mentioned
Feb 28, 2020	Tian S (China) (49)	Lobectomies	2	Edema, intra-alveolar fibrin, mononuclear inflammatory cells	Yes (“early DAD pattern” in 1 of 2)	None mentioned
April 10, 2020	Barton LM (USA) (46)	Complete autopsies	2	DAD, chronic airway inflammation	Yes (1 case)	Few (lung, 1 case)
April 11, 2020	Karami P (Iran) (47)	“Autopsy of lungs”	1	Hyaline membranes, viral cytopathic effect	Yes (hyaline membrane noted)	None mentioned
April 14, 2020	Tian S (China) (50)	Post-mortem biopsies of lung, liver, heart	4	DAD	Yes	None mentioned
April 15, 2020	Magro C (USA) (48)	Limited autopsies (2), skin biopsies (3)	5	“Hemorrhagic pneumonitis” (lung), “thrombogenic vasculopathy” (skin)	Yes (hyaline membranes in 1 of 2 cases in which lungs were examined)	Yes (skin)
April 16, 2020	Barnes BJ (USA) (45)	Autopsies (brief mention)	3	“Neutrophil extracellular traps”	Not mentioned	Nonementioned
April 20, 2020	Varga Z (Switzerland) (51)	Autopsies (2), small intestine resection (1)	3	Endotheliitis, DAD, viral inclusions in endothelial cells in kidney	Yes	“Only scattered fibrin thrombi”

*DAD, Diffuse alveolar damage*.

This publication was followed by a few small autopsy series (including “limited autopsies”) and another small series of post-mortem biopsies from China (48, 50, 51).

**Figure 5:**
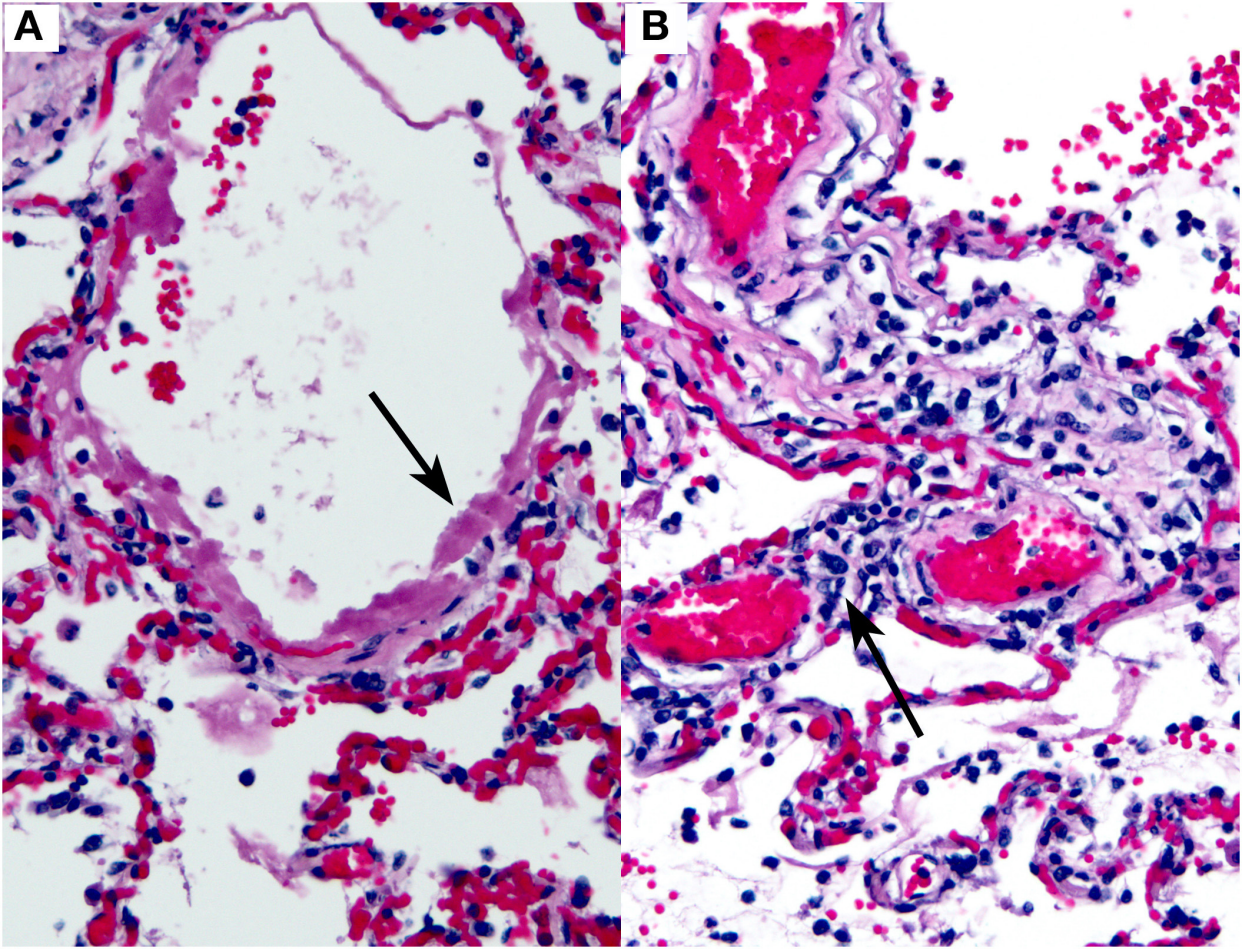
COVID-19 lung autopsy specimen. This figure is original and based on data from (46). It demonstrates COVID-19 pathology as seen in the lungs of an autopsied case (case 1, Barton et al.). **(A)** Diffuse alveolar damage. The arrow points to a hyaline membrane. **(B)** Interstitial lymphocytic inflammatory infiltrate. The arrow indicates lymphocytes within an alveolar septum. Hematoxylin-eosin stain, 200×, both images.

Thus, far, the most consistently reported finding in COVID-19 has been diffuse alveolar damage (DAD) in the lungs (Figure 5A). This finding has been observed in virtually every published case report or series thus far [Table 2; (46–52)]. DAD is a pathologic manifestation of severe acute lung injury. It is characterized by the presence of hyaline membranes in the acute stage and interstitial edema and fibroblast proliferation in the organizing stage. We would like to emphasize that DAD is not specific for COVID-19 but has a large list of potential causes, including shock, sepsis, severe trauma, other infections, connective tissue disease, drug toxicity, and toxic inhalants, among others (53–56). A subset of cases is idiopathic (57). Common secondary pathologic findings in DAD (regardless of etiology) include large, prominent and sometimes atypical type II pneumocytes, squamous metaplasia, and occasional thrombi within small pulmonary arteries. This last point is worth stressing: thrombi in the lung are well-known as a common secondary finding in DAD. They are thought to result from endothelial damage, which is central to the pathogenesis of DAD regardless of etiology. We stress this point to prevent misinterpretation of occasional thrombi in small arteries in the lung in the context of DAD as evidence of a more generalized thrombotic tendency. In fact, prominent thrombi were reported in lungs infected by H1N1, and at the time it was suggested that this finding might be unique to H1N1 (58).

Inflammatory infiltrates of various types have also been reported in COVID-19, including lymphocytic infiltrates in the airways and interstitium (46, 49), and neutrophils (45). An example of interstitial lymphocytic inflammatory infiltrates in the lung in a COVID-19 case is shown in Figure 5B.

**Figure 6:**
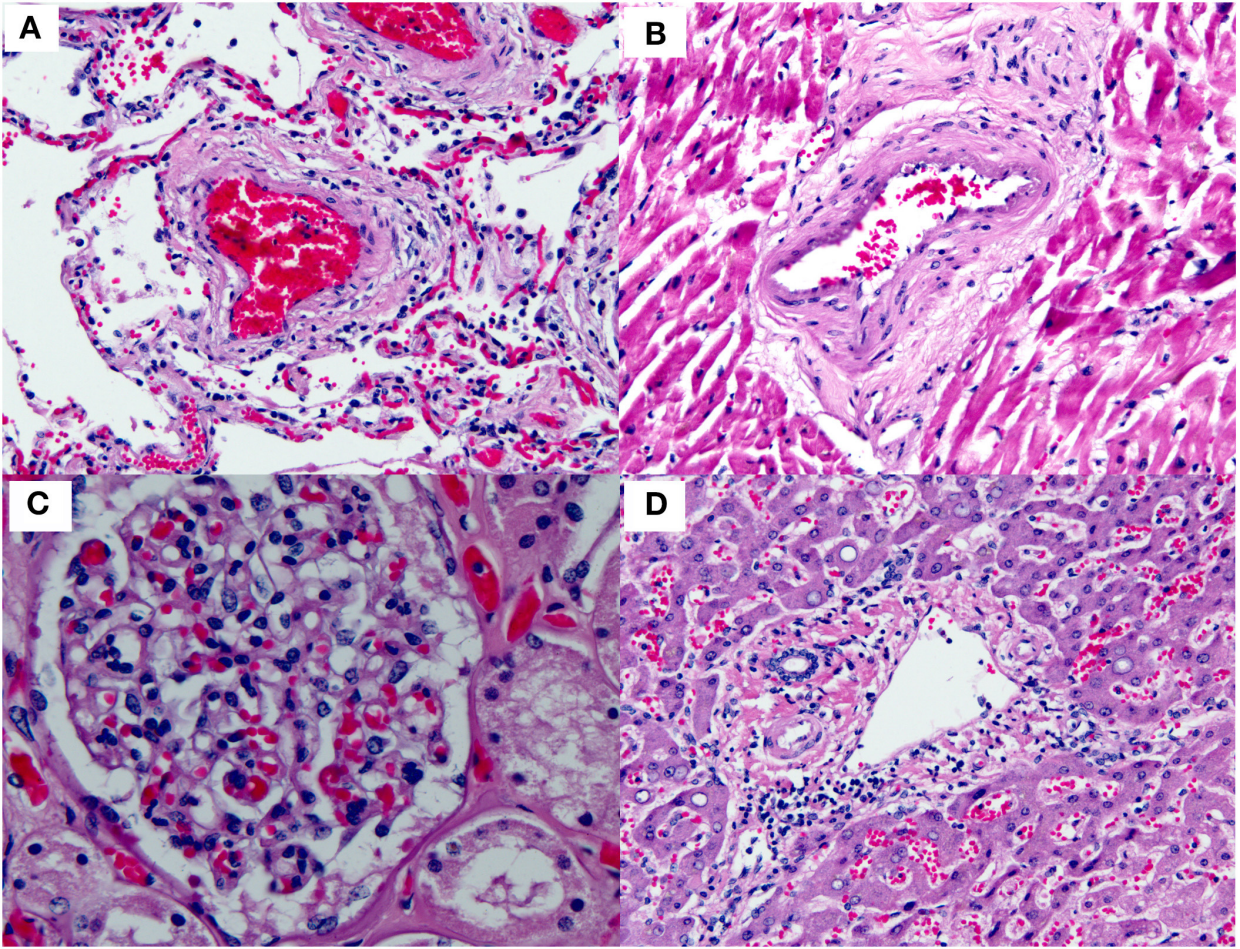
Small blood vessels in various organs in COVID-19. This figure is original and based on data from (46). No thrombi are seen in the small blood vessels of the **(A)** Lung. **(B)** Heart. **(C)** Kidney (glomerulus). **(D)** Liver (portal tract) (autopsy case 1, Barton et al.).

There has been intense clinical interest around the development of thrombi in a subset of patients with COVID-19. Interestingly, a widespread thrombotic process has not been documented in the majority of pathology specimens examined thus far (Figure 6 and Table 2). Only one report has illustrated a few thrombi in skin biopsies from three patients who presented with a purpuric rash. No published pathology reports have illustrated widespread multi-organ thrombi in the setting of COVID-19. In future pathology studies, it will be interesting to determine whether the clinical suspicion of widespread “microthrombosis” is confirmed by histopathology, and if so, to determine whether this is common or occurs only in a small subset of cases.

Other pathologic findings reported only sporadically in COVID-19 include viral inclusions, edema, intra-alveolar fibrin, and endotheliitis. To our knowledge, there are no reports of histologically documented myocarditis clearly attributable to COVID-19 thus far. Additionally, there is no evidence that any of the pathologic findings discussed above are pathognomonic of COVID-19.

## Clinical Symptoms

Clinical symptoms have been shown to occur most commonly between days 4 and 5 from exposure; however, studies have shown that the incubation period can last up to 14 days (5, 59). The most common symptoms reported in the literature so far include fever, cough, fatigue and shortness of breath, which are similar to other viral infections including the seasonal flu. One study identified 24 critically ill patients from nine Seattle-area hospitals with laboratory-confirmed COVID-19 infection with symptoms beginning 7 ± 4 days before admission. The most commonly reported symptoms were cough and shortness of breath and around 50% of patients had fever on admission (60). A case series study in New York, the epicenter of the pandemic in the U.S., that included 5,700 patients with COVID-19 infection found that 30.7% of the patients were febrile on admission (61). Another large study in China that extracted data from 1,099 patients with laboratory-confirmed COVID-19 showed that 43.8% of the patients had a fever on admission while 88.7% of patients developed a fever during their hospital stay. The second most commonly reported symptom was cough (67.8%) while fewer patients reported gastrointestinal symptoms such as nausea (5%) and diarrhea (3.8%) (59) (Table 3).

**Table 3:** A list of the most common clinical symptoms of SARS-CoV-2 infection based on a 1,099 patient study in China (59).

**Symptoms**	**Percentage (%)**
Fever	88.7
Cough	67.8
Fatigue	38.1
Sputum production	33.7
Shortness of breath	18.7
Myalgia or arthralgia	14.9
Sore throat	13.9
Headache	13.6
Chills	11.5
Nausea or vomiting	5
Nasal congestion	4.8
Diarrhea	3.8

Anosmia and dysgeusia have also been reported in patients with SARS-CoV-2 infection. A cross-sectional survey study found that these symptoms were frequently reported in patients infected with SARS-CoV-2 and, in most cases, preceded the onset of other symptoms (62). Asymptomatic infection has also been discussed in the literature; however, the frequency remains unclear. A study of 55 asymptomatic carriers with confirmed SARS-CoV-2 infection on admission found that the majority of these patients ended up having mild symptoms and a mild disease course while asymptomatic infection was rare and was mainly in young patients between 18 and 29 years of age (63). Another study involving 634 patients infected with COVID-19 on a cruise ship in Japan found that 17.9% were asymptomatic (64).

## Diagnosis

SARS-CoV-2 RNA is detected via reverse-transcription polymerase chain reaction (RT-PCR) most commonly collected from nasopharyngeal (NP) swabs. In the United States, the CDC recommends the collection of NP swabs for asymptomatic individuals. Instead, specimens from symptomatic patients should be collected from bilateral anterior nares and mid-turbinate. An oropharyngeal (OP) swab could be collected if an NP swab is not possible. The CDC also recommends collecting sputum in patients with a productive cough, however sputum induction is not recommended. Also, when clinically indicted (i.e., patients who are mechanically intubated), a lower respiratory tract sample via a bronchioalveolar lavage (BAL) should be collected (65).

The accuracy of SARS-CoV-2 testing is yet to be established. It has been noted that RT-PCR testing for SARS-CoV-2 could be falsely negative either due to insufficient viral load if the specimen is collected too early or too late in the disease course, or due to technical errors like being handled or shipped improperly (64, 66). There have been cases reported of patients presenting with classic computed tomography (CT) chest findings (bilateral peripheral distribution with multifocal lower lung involvement) combined with high clinical suspicion for SARS-CoV-2 infection who test negative on RT-PCR (67). Lower respiratory tract samples (i.e., BAL) are more likely to yield a positive result compared to upper respiratory tract samples. In a study involving 205 patients, 93% of BAL specimens (14 out of 15) were positive compared to 72% of NP swab specimens (72 out of 104) (68). Consequently, if initial testing is negative but clinical suspicion remains high, the WHO recommends repeat testing, preferably from a lower respiratory tract specimen, if possible.

Given that SARS-CoV-2 is a newly discovered virus, the antibody response in COVID-19 patients remains largely unknown. As of now, RT-PCR-based viral RNA is the current reference standard diagnostic tool for COVID-19 infections, but several studies are suggesting the incorporation of serologic antibody testing to aid in diagnosis of COVID-19 infections. These can be particularly useful in suspected patients with negative RT-PCR-based viral RNA and those with asymptomatic infections. In addition, these tests may improve the sensitivity of COVID-19 pathogenic diagnosis when combined with RT–PCR-based viral RNA testing.

In a study conducted by Zhao et al., among 173 patients with SARS-CoV-2 infection, the median seroconversion time for total antibodies, immunoglobulin-M (IgM), and immunoglobulin-G (IgG) against SARS-CoV-2 were day-11, day-12 and day-14, respectively. The presence of antibodies was <40% among patients within 1-week since onset, and rapidly increased to 100.0% for total antibodies, 94.3% for IgM, and 79.8% for IgG on day 15 after onset. In comparison, RNA detectability decreased from 66.7% in samples collected before day 7–45.5% during days 15–39 (69).

Another study by Long et al. showed that among 285 patients with COVID-19 infections, 100% of patients tested positive for antiviral IgG within 19 days after symptom onset. Within the same study, 4 out of 52 suspected cases with negative RT-PCR-based viral RNA for SARS-CoV-2 tested positive for virus-specific IgG or IgM (70).

Rapid point-of-care testing for SARS-CoV-2, which is also an IgG/IgM based test with a time to result of 20 min has shown a good specificity of 88.9% but low sensitivity of 36.4% making it a less effective test for screening (71). Despite their aid in diagnosis, antibody tests do impose limitations, especially as single screening tools since the sensitivity and specificity of serologic antibody tests are highly variable. Also, it might take several days from the onset of infection for the body to formulate these antibodies.

Similar to SARS-CoV and MERS-CoV, SARS-CoV-2 has been detected in blood and stool. Therefore, blood and stool specimens could be tested to aid with the diagnosis (66).

## Laboratory Findings

Hospitalized patients with SAR-CoV-2 infection have been found to have varying white blood cell counts. A study by Huang et al. showed leukopenia (< 4 × 10^9^ per L) in 25% of patients, normal leukocyte counts (4–10 × 10^9^ per L) in 45% of patients, and leukocytosis (> 10 × 10^9^ per L) in 30% of patients. Lymphopenia (< 1 × 10^9^ per L) was found in 63% of patients (35). Another study by Guan et al. showed that leukopenia was present in 33.7% of patients on admission and 36.2% of the cases had thrombocytopenia (59). In a systematic review and meta-analysis of 43 studies involving 3,600 patients, the most common laboratory abnormalities included elevated C-reactive protein (68.6%), lymphopenia (57.4%), and elevated lactate dehydrogenase (LDH) (51.6%) (72). A study done by Zhou et al. showed that elevated levels of LDH, serum ferritin, IL-6, and high sensitivity cardiac troponin I were all associated with worsening illness and higher mortality (73).

One of the most common laboratory findings in hospitalized patients with COVID-19 is an increased d-dimer level. In a large retrospective analysis study of 1,099 patients with confirmed COVID-19 in China, patients with more severe illness were more likely to have an elevated d- dimer level compared to patients with non-severe illness (59). In another retrospective analysis study of 183 patients with confirmed COVID-19 pneumonia in Wahun, non-survivors were found to have significantly higher d-dimer and fibrin degradation product (FDP) levels, and longer prothrombin time (PT) on admission compared to survivors. Fibrinogen and antithrombin (AT) levels were also significantly lower in non-survivors. Also, 71.4% of non-survivors had overt disseminated intravascular coagulation (DIC) during their hospitalization compared to only 0.6% of survivors. The results imply that abnormal coagulation parameters during COVID-19 pneumonia were significantly associated with poor prognosis (74). Studies also showed that blood urea nitrogen and creatinine levels progressively increased in critically ill patients (75).

## Radiological Findings

**Figure 7:**
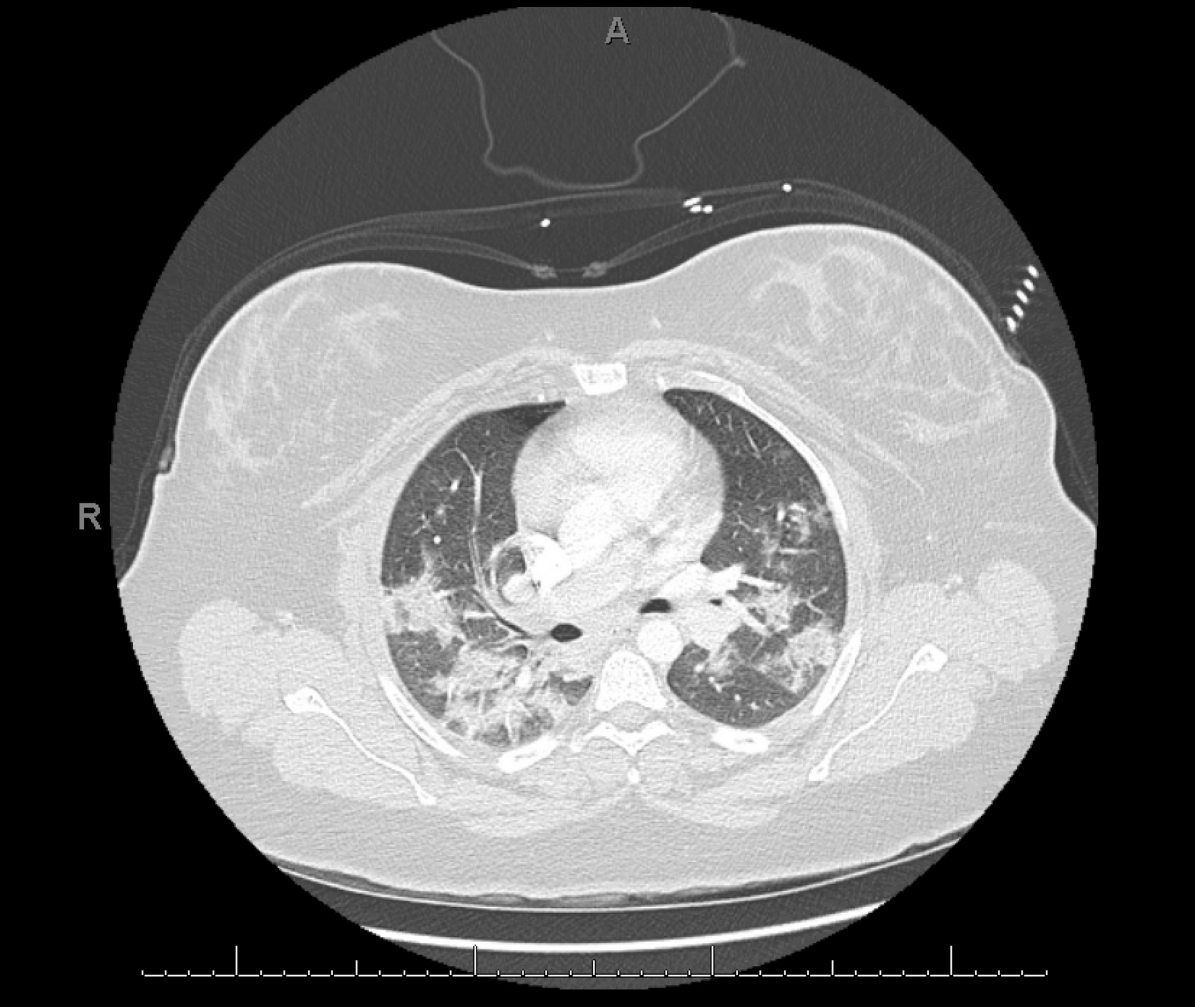
COVID-19 positive patient chest computed tomography (CT). This figure is original and illustrates the findings from (76). It demonstrates bilateral, predominately peripheral, patchy ground-glass opacities consistent with multi-lobar pneumonia.

Chest CT abnormalities during the early stages of COVID-19 are usually peripheral and focal or multifocal ground-glass opacities affecting both lungs in ~50–75% of patients (Figure 7). As the disease progresses, crazy paving and consolidation become the dominant CT findings, peaking around 9–13 days followed by slow clearing at ~1 month and beyond. Up to 50% of patients with COVID-19 infection may have normal chest CT scans 0–2 days after the onset of symptoms (76). On the other hand, it has been shown that abnormal chest CT findings may develop in asymptomatic patients (77). In one study, chest CT images from patients with SARS-CoV-2 who were admitted to the hospital showed some level of abnormality in all patients and bilateral lung involvement in around 98% of patients (40 out of 41) (35). Another study showed 86.2% of chest CT images on COVID-19 positive patients were abnormal and only 17.9% of patients had normal chest CT images, all of whom had mild disease (59).

**Figure 8:**
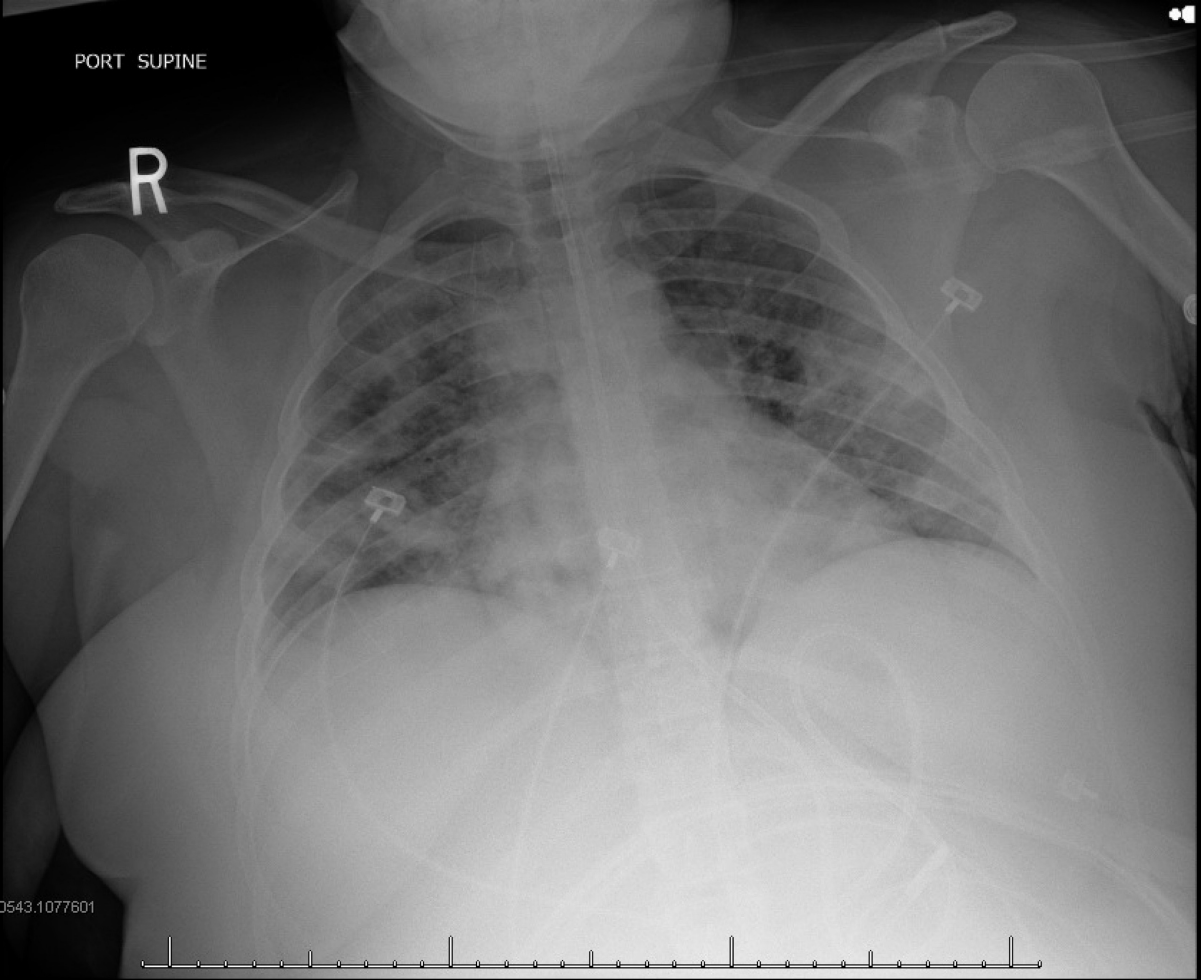
COVID-19 positive patient chest x-ray (CXR). This figure is original and illustrates the findings from (78). It demonstrates bilateral predominately mid to lower lung field airspace opacities.

During pandemics, physicians rely more on portable chest x-ray (CXR) since it is widely available and creates less exposure risk for staff compared to CT. However, some studies have shown that CXR may lack sensitivity for the detection of some lung changes frequently seen in COVID-19, which are otherwise detected with CT. Similar to CT, the most common reported CXR findings in COVID-19 include ground-glass opacities and lung consolidation (Figure 8) (78).

## Spectrum of Illness Severity and Course of Disease

The spectrum of illness associated with SARS-CoV-2 infection ranges from mild to severe and even fatal infection. The largest study to date was done by China CDC, which included around 44,672 patients with confirmed SARS-CoV-2 infection. This study showed that among 44,415 patients, the majority of the cases (81%) were classified as mild disease (i.e., mild pneumonia or no pneumonia) while ~14% were classified as severe disease (i.e., dyspnea with respiratory rate ≥30/min, blood oxygen saturation ≤93%, partial pressure of arterial oxygen to fraction of inspired oxygen ratio <300, and/or the development of diffuse lung infiltrates involving more than 50% of the lungs within 24–48 h), and 5% as critical disease (i.e., respiratory failure, shock, and/or multi-organ failure) (79).

The majority of patients with SARS-CoV-2 infection have been found to start with mild symptoms and, during the course of a week, progress to moderate or severe disease. A study done in Wuhan showed that, in the majority of patients, the median time to the development of dyspnea was 5 days, to hospital admission was 7 days, and to the development of ARDS was 8 days from the start of illness (75). Another study showed that the median time to mechanical ventilation was around 14.5 days from the onset of illness (73).

## Disease Complications

ARDS is one of the major complications of SARS-CoV-2 infection. A study involving 138 patients in Wuhan, China showed that 19.6% of the patients developed ARDS. Other common complications identified in this study included shock (8.7%), arrhythmia (16.7%), and acute cardiac injury (7.2%) (59). Patients who were admitted and received care in the ICU were more likely to develop these complications than non-ICU patients (75).

Another study including 191 patients in Wuhan, China showed that the most common complication was sepsis (59%) followed by respiratory failure (54%), ARDS (31%), heart failure (23%), and septic shock (20%). Other less frequent complications included coagulopathy (19%), defined as 5-s extension of activated partial thromboplastin time or 3-s extension of prothrombin time, and acute cardiac injury (17%), defined as elevated high sensitivity cardiac troponin I to above the 99th percentile of the upper reference limit or new EKG and/or echocardiogram findings. Non-survivors suffered more of these complications compared to survivors (73).

Interestingly, cardiac events such as new or worsening congestive heart failure, myocardial infarctions, arrythmias, and cardiac arrest occurred more frequently in patients with associated pneumonia (73).

In severe COVID-19 disease, hypercoagulability can be stimulated by endothelial cell dysfunction, increased blood viscosity from hypoxia, or hypoxia-induced transcription factor-dependent signaling pathway (80, 81). Acute venous thromboembolism (VTE) has been reported in patients with SARS-CoV-2 infection. A Dutch study involving 184 ICU with proven COVID-19 found a 31% incidence of thrombotic complications, of which 27% comprised of radiographically confirmed VTE. Pulmonary embolism (PE) was the most frequent of these thrombotic complications (82). Another study in Wuhan, China showed that 66 out of the 143 hospitalized patients with COVID-19 included in the study developed a lower extremity deep vein thrombosis (DVT). Their analysis suggested multifactorial causes of DVT in these patients including older age, more severe illness, more chronic illness, stasis, and high thrombotic and inflammatory abnormalities (83). A case report by Danzi et al. described the case of a 75-year-old COVID-19 positive hospitalized female radiographically diagnosed with a pulmonary embolism who had no other predisposing factors other than the acute infection with COVID-19 (84).

## Risk Factors Associated With Severe Disease

Many studies have shown that severe illness and death occur in patients with certain risk factors including older age and underlying medical comorbidities. A study done by Wu et al. showed that among 44,672 cases of COVID-19 in Wuhan, China, the majority of patients were 30 to 79 years of age (87%) followed by those aged 80 years and older (3%) while only 1% were aged 9 years and younger (79). Older age was one of the identified risk factors associated with poor prognosis and death (73). A study by Guan et al. showed that those with severe disease were older by a mean of 7 years compared to those with mild disease (59).

It remains unclear whether gender is an independent risk factor for more severe disease. A retrospective case series done in New York, showed that among the 393 patients with confirmed COVID-19, 60.6% were males. Also, males were more likely to receive mechanical ventilation (85). However, this correlation does not imply causation since this study did not adjust for other medical comorbidities.

A study by Guan et al. showed that patients with severe disease were more likely to have an underlying coexisting illness compared to those with non-severe disease (38.7 vs. 21%) (59). Another study done in Wuhan, China showed that among 191 patients with COVID-19, hypertension (30%) was the most commonly reported comorbidity followed by diabetes (19%), coronary heart disease (8%), and chronic obstructive lung disease (3%) (73). According to data from the CDC in the US, among 7,162 patients with reported medical problems, diabetes mellitus (10.9%), chronic lung disease (9.2%), and cardiovascular disease (9.0%) were the most commonly reported comorbidities. Immunocompromising conditions (3.7%) and chronic kidney disease (3%) were also reported (86). In a case series study in New York including 5,700 patients with COVID-19 infection, the most common comorbidities in hospitalized patients were hypertension (56.6%), obesity (41.7%), and diabetes (33.8%) (61). Obesity was found to be risk factor for intubation in a retrospective cohort study of 124 patients with SARS-CoV-2 infection. Of the patients who were intubated, 47.6% had a body mass index (BMI) > 30 kg/m^2^ and 28.2% had a BMI > 35 kg/m^2^ (87).

## Case Fatality Rates

Case Fatality Rates (CFR) is defined as the ratio between confirmed deaths and confirmed cases. To date, SARS-CoV-2 seems to have a lower CFR compared to SARS-CoV and MERS-CoV (Table 1).

Estimating the CFR in an ongoing pandemic can be challenging since it is subject to considerable change as more cases emerge and more outcomes unveil. CFR varies depending on multiple factors including testing strategies. For example, low testing capability can lead to an over-estimation of the CFR by causing an under-estimation of the number of confirmed cases (14, 88).

China CDC estimated the CFR to be around 2.3% among 44,672 confirmed COVID-19 cases, 8% of whom were aged 70–79 years, 14.8% aged 80 years and older, and 0% were among those aged 9 years or younger (79).

According to the Italian National Institute of Health, the CFR in Italy was 7.2% among 22,512 cases up to March 17, 2020 (14). According to data collected by the South Korea CDC, the CFR was 1.79% among 10,237 cases up to April 5, 2020 (89). According to the CDC in the United States, the CFR was 2.5% among 304,826 cases as of April 5, 2020 (65).

The numbers of cases and deaths are evolving on a daily basis; however, it remains unclear why there is such a big difference in CFR across different countries. As noted, the overall CFR in Italy is significantly higher than that reported in China (2.3 vs. 7.2%). The demographic characteristics of the Italian population in 2019 showed that ~23% of its population was above the age of 65. This might somehow explain Italy's higher CFR compared to other countries affected by the virus with smaller proportions of their populations in this age group.

However, when data was stratified according to age groups, the CFR in Italy and China were similar among those aged 0–69 years but the CFR remained significantly higher in Italy compared to China in patients aged 70 years and older (14). Understanding this significant difference in CFR across countries remains challenging and further studies are required to comprehend it fully.

## Investigational Approaches and Adjunctive Therapies

Unfortunately, up until this point, there has yet to be a vaccine or proven effective therapy against SARS-CoV-2 infection. While many trials, including much needed randomized controlled trials (RCTs), are currently underway, the mainstay of therapy remains supportive care. This ranges from symptomatic treatment to ventilator support for patients with ARDS depending on illness severity. This also includes recognizing and treating superimposed bacterial infections and/or sepsis early on. Many of the current clinical trials are investigating drugs that were previously used to treat SARS-CoV and MERS-CoV. These will be discussed further below.

### Chloroquine/Hydroxychloroquine

Chloroquine and hydroxychloroquine are widely used anti-malarial drugs. Hydroxychloroquine is a chloroquine analog with less drug to drug interaction and a better safety profile.

Both chloroquine and hydroxychloroquine are shown to inhibit the growth of SARS-CoV-2 *in vitro* and decrease viral replication in a concentration-dependent manner. Hydroxychloroquine was found to be more potent. It has been hypothesized that both chloroquine and hydroxychloroquine may inhibit SARS-CoV-2 replication. They may do this by changing the pH at the surface of the cell membrane thereby inhibiting fusion in addition to inhibiting nucleic acid replication, glycosylation, and viral assembly and release (90).

Multicenter clinical trials in China showed that chloroquine was effective and had an acceptable safety profile in patients with SARS-CoV-2 pneumonia (91). Hydroxychloroquine is currently under investigation in various RCTs in the Unites States for treatment in patients with SARS-CoV-2 infection and also for pre-exposure and post-exposure prophylaxis. In one retrospective cohort study involving 1,438 patients hospitalized in metropolitan New York, treatment with hydroxychloroquine, azithromycin, or both was not associated with significantly lower in-hospital mortality when compared to neither treatment. However, the interpretation of these findings may be limited by the observational design (92).

In another randomized, double-blind, placebo-controlled trial in the US, 821 asymptomatic participants were randomly assigned to receive either placebo or hydroxychloroquine 4 days after exposure to someone with confirmed COVID-19. The study found that hydroxychloroquine did not prevent illness related to COVID-19 or confirmed infection when used as postexposure prophylaxis within this timeframe (93).

On June 15, 2020, the U.S. Food and Drug Administration (FDA) revoked the emergency use authorization granted on March 28, 2020 for chloroquine phosphate and hydroxychloroquine sulfate in certain hospitalized COVID-19 patients. They cite the serious cardiac adverse events and other protentional serious side effects to outweigh the potential benefits of their use (94).

### Azithromycin

Azithromycin is a macrolide antibiotic that has been widely used in patients with chronic pulmonary inflammatory disorders and/or community acquired pneumonia for its anti-inflammatory effect (95). However, there is limited data suggesting the beneficial effect of azithromycin in combination with chloroquine/hydroxychloroquine in the treatment of ARDS in patients with SARS-CoV-2 infection.

An open-label non-randomized clinical trial of 36 patients done in China showed a synergistic effect combining hydroxychloroquine and azithromycin in treatment of SARS-CoV-2 infection by reducing the detection of SARS-CoV-2 RNA in specimens from the upper respiratory tract (96). However, this study did not comment on the clinical benefit of this combination. Another small observational study in China showed that combining hydroxychloroquine and azithromycin for the treatment of SARS-CoV-2 in hospitalized patients had no clinical benefit and no evidence of rapid viral RNA clearance (97). Hydroxychloroquine and azithromycin can both lead to corrected QT (QTc) prolongation, which can lead to fatal arrythmias. Therefore, they should be used with caution in patients with prolonged QTc and those with certain medical conditions such as hepatic or renal disease.

### Remdesivir

Remdesivir is a novel nucleotide analog that incorporates into nascent viral RNA chains and causes premature termination inhibiting viral replication. Remdesivir has been shown to be an effective antiviral agent against beta-coronaviruses such as SARS-CoV and SARS-MERS in mice, non-human primates and *in vitro*, and is currently in clinical trials for the treatment of Ebola virus (98).

A study in China showed that remdesivir is highly effective in controlling SARS-CoV-2 infection *in vitro* (98). Another study that was recently published involving compassionate-use of remdesivir showed clinical improvement in 68% of patient (36 out of 53) who had severe SARS-CoV-2 infection; 57% were extubated and 47% were discharged (99).

Despite its promising results *in vitro, in vivo* in animal models, and in compassionate-use studies in humans, remdesivir is still not approved by the FDA for use as a standard of care therapy due to lack of established data on safety and efficacy in humans. The biopharmaceutical company Giliad has initiated two phase 3 clinical trials to evaluate the safety and efficacy of this drug in COVID-19 patients.

### Lopinavir-Ritonavir

Lopinavir-ritonavir is a protease inhibitor combination that has been used against human immunodeficiency virus (HIV) infection. This drug was proven to have *in vitro* activity against SARS-CoV; however, it does not seem to have a clear benefit during the current outbreak (100). A randomized, controlled, open-label trial that included 199 patients assessed the use of lopinavir–ritonavir treatment in patients with SARS-CoV-2 and showed no benefit with administration of the drug compared to standard care alone, which comprised of antibiotics, vasopressors, renal replacement therapy, extracorporeal membrane oxygenation (ECMO) and/or supplemental oxygen/invasive ventilation if needed. Gastrointestinal adverse events were higher in the lopinavir–ritonavir group compared to those receiving standard-care alone; however, adverse events were higher in the standard-care group overall (101).

### Favipiravir

Favipiravir is an RNA polymerase inhibitor that is used for the treatment of influenza in China. Favipiravir is able to block the replication of RNA viruses by blocking the RNA-dependent RNA polymerase (RdRp) enzyme. Therefore, favipiravir may have antiviral activity against SARS-CoV-2, which is also an RNA virus (102). Clinic trials involving the use of this drug in treating SARS-CoV-2 infection are currently ongoing.

### IL-6 Pathway Inhibitors

As previously mentioned, cytokine storm syndrome and increased levels of IL-6 have been described in patients with severe SARS-CoV-2 infection. IL-6 levels were found to be 2.9-fold higher in patients with severe complicated SARS-CoV-2 infection, including those with ARDS, when compared to mild, non-complicated disease. Until now, there are no RCTs showing that IL-6 inhibitors benefit patients with SARS-CoV-2 infection. However, preliminary investigation demonstrated that IL-6 inhibitors are safe and efficacious in these patients. A single non-randomized, single-arm study showed that patients with severe SARS-CoV-2 infection who received tocilizumab, an IL-6 inhibitor, showed significant clinical improvement including decreased oxygen requirement and resolution of radiographic abnormalities (37).

Treatment guidelines from China's National Health Commission included tocilizumab for patients with severe SARS-CoV-2 infection who also have increased IL-6 levels based on a multicenter, randomized controlled trial (103). Multiple IL-6 inhibitors including tocilizumab, sarilumab, and siltuximab are currently under investigation in clinical trials in China.

### Ivermectin

Ivermectin is an FDA-approved medication for the treatment of various parasites and has an established safety profile in humans. Ivermectin has been shown to inhibit *in vitro* replication of various positive single stranded RNA viruses such as dengue and west Nile (104, 105). This drug has recently demonstrated *in vitro* activity against SARS-CoV-2 when a single dose was able to control viral replication within 24–48 h. It is hypothesized that this is likely through the inhibition of importin α/β1 heterodimer, which mediates nuclear import of viral proteins, a process that many RNA viruses rely on during infection (105, 106). The FDA has not yet approved ivermectin for the prevention or treatment of SARS-CoV-2 infection. RCTs studying the efficacy and safety of this drug in COVID-19 are still lacking.

### Corticosteroids

The use of glucocorticoids in patients with SARS-CoV-2 infection, especially in those with severe disease, was a point of major controversy. The rationale behind their use is to decrease lung inflammation as seen in ARDS. However, this comes with adverse effects such as inhibiting the immune response and thus increasing the risk of secondary infections as well as delaying viral clearance (107). A Cochran review published in July 2019 that included 48 RCTs found insufficient evidence to determine if corticosteroids were effective at reducing mortality and duration of mechanical ventilation in patients with ARDS (108).

A recent randomized, controlled, open label study known as the RECOVERY trial included 2,104 COVID-19 patients in the United Kingdom (UK) who were randomly allocated to receive 6 mg of dexamethasone per day for up to 10 days compared to standard of care therapy alone. Preliminary results from this trial showed that dexamethasone use reduced 28-days mortality among those with severe disease (i.e., those receiving invasive mechanical ventilation or oxygen support) but not among patients with mild disease (i.e., those who did not receive any respiratory support) (109).

Prior to this trial, many treatment guidelines stated that corticosteroids were either not recommended or contraindicated in COVID-19 patients. The WHO welcomed the preliminary results of the RECOVERY trial and will soon be updating their guidelines regarding how and when dexamethasone should be used in COVID-19 patients (110).

### Convalescent Plasma

Convalescent plasma (CP) therapy is a classic adaptive immunotherapy that has been used for decades in the prevention and treatment of various diseases. CP was used in prior epidemics including SARS-CoV, MERS-CoV, and H1N1 in 2009 and it showed successful results with a safe profile (111). Given the similarity between SARS-CoV-2, SARS-CoV, and MERS-CoV, CP may have potential efficacy in this current pandemic. However, no RCTs involving CP in SARS-CoV-2 infection have been completed as of yet, and hence the risks and benefits remain unclear.

In an uncontrolled case series, the treatment of five patients with severe SARS-CoV-2 infection and ARDS with CP showed clinical improvement in all five cases. All of these patients showed stabilization in their vital signs, decrease in inflammatory biomarkers (CRP, IL-6 and procalcitonin), and improvement of abnormalities on imaging. Three out of five of these patients were successfully extubated (112). Another study showed that the use of CP in 10 patients with severe SARS-CoV-2 infection resulted in significant clinical improvement with no side effects. All patients had disappearance of viremia within 7 days, improvement in their clinical symptoms, and improvement in their chest radiographic abnormalities (111).

In the United States, the FDA is accommodating emergent investigational application for the use of CP in patients with severe or immediate life-threatening SARS-CoV-2 infection, such as those in respiratory failure, septic shock and/or multiorgan failure (113).

### Heparin

As more studies emerge linking coagulopathies to COVID-19 including systemic thrombosis and DIC, this raises the question whether heparin should be used in hospitalized patients to prevent these complications.

In a retrospective study in China that included 449 patients, patients who received a prophylactic dose of heparin when they had sepsis-induced coagulopathy (SIC) score ≥ 6 and a d-dimer level >6-fold of upper limit of normal had decreased mortality (81). Based on the limited available data, the International Society of Thrombosis and Hemostasis (ISTH) recommends the measurement of d-dimer, PT, and platelet count for all patients with COVID-19 infection to help with risk stratification. The society also recommends the administration of low molecular weight heparin at prophylactic dose to all hospitalized patients with no contraindications (114). RCTs examining the use of heparin in COVID-19 patients are required to make appropriate recommendations.

### Vitamin C

Vitamin C, also known as ascorbic acid, has antioxidant properties and plays a significant role in reducing inflammatory response. Studies have shown that ascorbic acid down-regulates the production of pro-inflammatory cytokines (115). These concepts have generated interest in the use of ascorbic acid in the management of inflammatory conditions. In a recent randomized clinical trial involving 167 patients in the intensive care unit, intravenous infusion of high-dose ascorbic acid compared to placebo did not significantly reduce organ dysfunction scores or improve levels of biomarkers indicating inflammation among patients with sepsis and ARDS, two disease processes heavily associated with inflammation (116). A randomized controlled trial is currently underway and in phase 2 to study the clinical efficacy and safety of vitamin C infusion for treatment of COVID-19 pneumonia (117).

### Zinc

It has been shown that increased zinc concentration inside the cell can effectively impair replication of a number of RNA viruses such as influenza and polioviruses. A study showed that zinc in combination with zinc-ionophores like pyrithione inhibited the replication of SARS-CoV in cell cultures (118). Therefore, zinc supplementation may be of potential benefit for prophylaxis and treatment of COVID-19 and it is currently under investigation in multiple clinical trials in combination with other agents including hydroxychloroquine, vitamin C, and vitamin D (119).

### Montelukast

Montelukast has been shown to suppress oxidative stress and have anti-inflammatory effects. Use of high dose montelukast has been effective in the treatment of acute asthma. Because much of the morbidity and mortality from COVID-19 infection is due to excessive inflammatory processes, it is thought that montelukast may play a role in limiting the progression of disease (120). One of the protein complexes involved in cytokine production and inflammatory responses is NF-B (nuclear factor kappa-light-chain-enhancer of activated B cells). Therefore, inhibition of the NF-B signalizing pathway has been investigated for potential therapeutic options in inflammatory diseases. Montelukast inhibits the signaling of NF-B and other proinflammatory mediators. Its use in COVID-19 infection is currently being studied in a large clinical trial, which is in phase 3, compared with placebo (121).

### Potential Vaccines

To date, there is no vaccine proven effective against SARS-CoV-2 infection. There are numerous potential vaccines currently being investigated. The COVID-19 vaccine research and development landscape includes 115 vaccine candidates globally as of April 8, 2020. 78 of these candidates are confirmed, 73 of which are at exploratory or preclinical stages (122). One of the more advanced candidates that has recently moved into clinical development in the United States involves a messenger RNA platform (mRNA-1273), which encodes for the viral S protein of SARS-CoV-2 (123).

## COVID-19 Response

Many have criticized the global response to COVID-19 due to the rapidly increasing number of cases and deaths worldwide. It is important to highlight the sequence of events in this response in order to recognize areas of concern and associated consequences, and to extract potential lessons and improvements for future pandemics.

As previously mentioned, the cluster of cases identified in Wuhan were reported to the WHO by Chinese authorities on December 31, 2019 and confirmed to be associated with a novel coronavirus, later termed COVID-19, on January 8, 2020 (124). There have been multiple reports of suspected intimidation of clinicians who initially identified cases linked to COVID-19, which likely led to a delay in the release of information and a lack of transparency (125).

On January 17, consistent with existing communicable disease response protocols based on previous pandemics, the CDC introduced screening of travelers entering at 5 major US airports on direct and connecting flights from Wuhan, China. Travel bans were not instituted by the Chinese government until January 24, when they started restricting travel in and out of Hubei province (124). However, according to Wuhan officials, by the time these travel restrictions were instituted, 5 million people had already traveled from Wuhan to other locations for Lunar New Year (126). These restrictions were placed almost 1 month after the first cases of COVID-19 were detected. This delay in travel restrictions and continued ability of citizens traveling from high-risk areas to freely pass through international borders with minimal health screening allowed individuals potentially infected with COVID-19 to spread the infection both nationally and internationally (125).

As cases began to spread outside of Mainland China, on January 21, the CDC activated its Emergency Operations Center to optimize coordination for domestic and international COVID-19 response efforts. The WHO director-general declared that the COVID-19 outbreak constitutes a Public Health Emergency of International Concern (PHEIC) on January 30 (124). The International Health Regulations (IHR) grants the WHO director-general to declare a PHEIC for an extraordinary event that requires a coordinated international response as it poses a public health risk to other states through international spread. The WHO has previously declared five PHEICs: H1N1 in 2009, Polio in 2014, Ebola in West Africa in 2014, Zika in 2016, and Ebola in the Democratic Republic of Congo in 2019. This declaration is a powerful signal to the international community to launch a surge public health response and mobilize both political action and funding (126). This declaration acknowledging and widely broadcasting the severity of this outbreak came 1 month after the initial cluster of cases, possibly delaying appropriate containment measures (125).

Just 1 day later, on January 31, the secretary of the US Department of Health and Human Services (HHS) declared the response to COVID-19 a US public health emergency (124). This declaration authorizes enhanced federal powers, interjurisdictional coordination, additional resources, and waivers of specific regulations. The exercise of federal powers is based on the need to prevent dire public health, national security, economic, and societal consequences. The federal powers exercised by the HHS in the response to COVID-19 goes beyond those ever used for other public health emergencies such as Ebola, SARS, and H1N1 influenza. Following this declaration, federal agencies immediately implemented travel warnings, border protections, and entry bans (127). Since this declaration, multiple federal agencies, the CDC, and state and local health departments have also implemented other aggressive measures in an attempt to slow the spread of this illness, better prepare the health care systems for widespread transmission with considerable associated illness, and gain a better understanding of COVID-19 to guide public health recommendations and the development of diagnostics, therapeutics, and vaccines (124).

Perhaps, the most apparent and life-changing measure to the general public is the implementation of mitigation strategies, which are non-pharmaceutical interventions for communities with local transmission. These strategies are based on lessons learned from previous pandemics and are interventions that assist in slowing transmission of the virus in communities. This is an especially important feat prior to the wide availability of a pandemic vaccine. These strategies include “personal protective measures for everyday use” like self-isolation and hand hygiene; “personal protective measures reserved for pandemics” like home quarantine and wearing face masks when ill; “community measures aimed at increasing social distancing” like closing schools and stopping mass gatherings; and “environmental measures” like cleaning all surfaces that are frequently touched (128). The timing of the implementation of these strategies during the current pandemic has been under scrutiny.

## A Second Wave

As seen in multiple previous pandemics including the influenza pandemic of 1918, the first wave is often followed several months later by a second wave of infections that could potentially be even worse than the first. A second wave can be caused by a region being re-exposed to infection by an influx of infected people from another. The degree of the resulting new outbreak will depend on the level of immunity in the first region from the initial wave. This will be influenced by multiple factors including the potential for endogenous loss of immunity in the first population and the introduction of people who are not immune, for example, individuals moving from one state to another in the U.S. (129). To date, mitigation strategies have been effective at controlling the pandemic in several regions. A study by Aleta et al. showed that removing these restrictions could lead to a second wave of COVID-19 infections that could overwhelm the health care system. However, combining this with enhanced testing and contact tracing can reduce transmission and allow for reopening of economic activities, while having a manageable impact on the health care system even in the absence of herd immunity (130).

## Lessons Learned for Future Pandemics

As this pandemic continues to develop and continues to take the lives of so many, there are innumerable lessons to be learned for future pandemics. To begin with, it is crucial to establish clear whistleblowing policies for potential global health emergencies. This will allow for transparency and help encourage clinicians to bring important information to light as soon as they are detected. Once high-risk areas have been identified, precautions including travel restrictions and quarantines should be implemented as soon as a possible health threat is identified. Also, framework should be developed to escalate a threat status earlier for fast-spreading diseases (125). It is then crucial to implement population-based interventions including social distancing, quarantine, and isolation actions promptly. And finally, it is imperative for health care systems along with local, regional, and global forces to work together to ensure better preparedness for future pandemics in all aspects including staffing, supplies, the number of hospital beds, testing capacity, research and development, and policy. A high price was paid for these difficult lessons to be learned, so it is now our responsibility to dedicate the appropriate funding and efforts to prevent this level of catastrophe from repeating itself (131).

## Conclusion

Pandemics propose an immense challenge to public health, health care systems, and global economic security. Due to modern agricultural practices that increase human-animal interface, new zoonotic coronaviruses are likely to continue to spillover from animals to humans causing future outbreaks. Gaining insight into every aspect of coronaviruses is crucial to implement proper control measures to help prevent these outbreaks or lessen their impact on humans and society if they were to still happen. Special focus should be placed on understanding their pathophysiology to help better tailor and generate effective drug therapies and vaccinations. Nevertheless, our ability to handle future outbreaks will rely on the actions we take based on the lessons we have learned from previous pandemics. We hope that the rapidly developing research on the current COVID-19 pandemic will help provide the new information needed to fill these gaps.

## Author Contributions

NC, SC, and RB are the first, second, and third authors, respectively. They contributed equally to the literature search and the writing for the whole manuscript. AA contributed to the literature search and the preliminary writing for radiologic findings. AS and MR edited and commented on the final manuscript. SM, ES, ED, and LB contributed to the literature search and the writing of the histopathology section, in addition to providing original histopathology figures. IH is the senior and corresponding author. She provided the idea, supervised the entire process from inception to the final submission, and edited the final manuscript.

## Conflict of Interest

The authors declare that the research was conducted in the absence of any commercial or financial relationships that could be construed as a potential conflict of interest.
